# Prevention of influenza-related illness in young infants by maternal vaccination during pregnancy

**DOI:** 10.12688/f1000research.12473.1

**Published:** 2018-01-29

**Authors:** Marta C Nunes, Shabir A Madhi

**Affiliations:** 1 Medical Research Council, Respiratory and Meningeal Pathogens Research Unit, University of the Witwatersrand, Johannesburg, South Africa; 2 Department of Science and Technology/National Research Foundation, Vaccine Preventable Diseases, University of the Witwatersrand, Johannesburg, South Africa

**Keywords:** influenza, pregnancy, vaccination, maternal vaccination

## Abstract

The influenza virus circulates yearly and causes global epidemics. Influenza infection affects all age groups and causes mild to severe illness, and young infants are at particular risk for serious disease. The most effective measure to prevent influenza disease is vaccination; however, no vaccine is licensed for use in infants younger than 6 months old. Thus, there is a crucial need for other preventive strategies in this high-risk age group. Influenza vaccination during pregnancy protects both the mothers and the young infants against influenza infection. Vaccination during pregnancy boosts the maternal antibodies and increases the transfer of immunoglobulin G from the mother to the fetus through the placenta, which confers protection against infection in infants too young to be vaccinated. Data from clinical trials and observational studies did not demonstrate adverse effects to the mother, the fetus, or the infant after maternal influenza vaccination. We present the current data on the effectiveness and safety of influenza vaccination during pregnancy in preventing disease in the young infant.

## Introduction

Influenza is a contagious, acute viral illness of the respiratory tract. The World Health Organization (WHO) estimates that during normal seasonal epidemics 5–15% of the population is typically infected by influenza viruses and that there are 3 to 5 million cases of severe illness and up to 500,000 influenza-associated deaths per year globally ^1, 2^. The highest burden of severe illness is concentrated in those younger than 1 year old and older than 65 years old ^3, 4^. Neonates and infants are especially susceptible because they are naïve to past influenza virus infections and immunologically immature ^5– 8^.

Vaccination is the best way to prevent influenza illness. Safe and effective vaccines are available and have been used for more than 70 years in the general population ^9^. Nonetheless, efforts to protect infants from influenza infection during the first 6 months of life through direct vaccination have been unsuccessful with the available influenza vaccine formulations. Although safety was not a concern in the four published studies on inactivated influenza vaccines (IIVs) in infants younger than 6 months old ^10– 13^, immune responses to the vaccine were in general weak and attenuated possibly by the high levels of maternal antibodies ^10– 12^. Studies with live attenuated influenza vaccines in infants reported rates of seroconversion similar to those observed with IIV, but a higher number of adverse effects was reported ^14– 16^. Furthermore, vaccination schedules—with the exception of Bacille Calmette-Guérin, polio, and hepatitis B—start at only 6–8 weeks of age because of the inability of neonates to develop robust immunity ^17^. Generally, this has been attributed to the absence of a strong type 1 T-helper cellular response, cytotoxic responses, trends to immunoregulatory responses, and weak plasma cell and germinal B-cell responses early in life ^18^.

There is currently no influenza vaccine approved by regulators and recommended in any country for use in infants younger than 6 months old, even if they have high-risk conditions for which influenza vaccination is recommended among older children. However, infants can be protected from influenza illness in the first few months of life by maternal antibodies transferred via the placenta during pregnancy and possibly through breastmilk postpartum ^19– 22^. Therefore, efforts have turned to active vaccination during pregnancy as a means to provide neonates and young infants with passive immunity to influenza. Based on the substantial risk of severe disease in young infants and the safety of influenza vaccine during pregnancy, the WHO, the US Centers for Disease Control and Prevention, and other national public health organizations recommend that pregnant women be vaccinated at any stage during pregnancy and be given high priority for influenza vaccination ^23– 25^.

This review focuses on recent advances in the prevention of influenza illness in young infants (defined as less than 6 months of age) through vaccination during pregnancy. We also give a brief overview of the influenza viruses and epidemiology of the disease in this age group.

## Influenza viruses

Influenza is caused by influenza viruses that circulate worldwide and that can be spread from person to person through airborne droplets or contact. Influenza viruses belong to the orthomyxoviridae family and have a segmented, negative-sense, single-stranded RNA genome with helical symmetry and different sizes of ribonucleoproteins. The influenza virus genus is divided into three types—A, B, and C—as defined by the antigenicity of the nucleoproteins and matrix proteins in the viral core ^26^. Influenza A viruses are further divided into subtypes according to the antigenic properties of the surface glycoproteins hemagglutinin and neuraminidase. Eighteen antigenically different hemagglutinins (H1–18) and eleven different neuraminidases (N1–11) have been identified and their combination designates the virus subtype. Currently, H1 and H3 subtypes of influenza A are endemic in humans. Circulating influenza B viruses are classified into two groups: Yamagata-like or Victoria-like lineages ^27^. Influenza A and B viruses are responsible for seasonal epidemics, and current vaccines are designed to target these viruses. Influenza type C viruses are detected much less frequently and cause mild infections, which do not present significant public health concerns. Influenza types differ in the range of animal hosts that they can infect: humans are the only known reservoir of influenza B and C, whereas influenza A is also found in other animals ^28^. Given the multiple subtypes, higher mutation rates, and diverse hosts, influenza A poses the greatest pandemic threat. People are susceptible to influenza throughout their lives because of the ability of the virus to continually mutate by antigenic drift and antigenic shift (only influenza A viruses) mechanisms ^29^.

Influenza infection attack rates and disease severity vary considerably from season to season and across world regions. In temperate countries, influenza viruses typically circulate at higher levels during colder winter months; in many tropical and subtropical regions, influenza occurs throughout the year.

## Clinical presentation and disease burden

The majority of influenza virus infections are self-limited, and the common symptoms are fever, chills, sore throat, myalgia, headache, coughing, and general discomfort. Most people recover within a week without requiring medical attention ^30^. But influenza can cause severe illness or death, and infants are especially vulnerable to serious influenza complications ^31^. Infants younger than 6 months old have the highest burden of childhood complications and death associated with influenza ^32^. In severe pediatric cases, pneumonia, acute respiratory distress syndrome, secondary bacterial infection, febrile seizures, and neurologic complications such as encephalopathy and encephalitis can occur ^33, 34^. Influenza infection has also been associated with apnea in neonates ^35^. However, in neonates and young children, non-febrile or non-respiratory manifestations of influenza infection (or both) are common ^36, 37^. The presentation of unspecific clinical signs renders surveillance difficult; thus, the real burden of influenza infections among the younger population is challenging to estimate. Another challenge to truly describing the overall burden of influenza-related severe disease is that severe complications from influenza virus infection, such as pneumonia, may occur days after the primary viral infection, when current diagnostics are less sensitive to detect low viral load by the time of hospitalization and testing ^38^. Hence, the estimated rates of laboratory-confirmed influenza are likely to be an underestimation of the true burden of influenza disease ^39^.

In the USA, during some winters, up to 10% of all infants are brought in for medical care for influenza-associated illness, including hospitalization ^7, 40^. Data are more sparse from low- to middle-income countries (LMICs), but a recent systematic analysis on the burden of influenza in pediatric respiratory hospitalizations worldwide estimated that influenza causes approximately 870,000 hospitalizations per year in children younger than 5 years old (135/100,000 children per year), including 374,000 hospitalizations per year in infants younger than 1 year old (284/100,000 infants per year), 228,000 of which occur in infants younger than 6 months old ^1^. Furthermore, laboratory-confirmed influenza-associated hospitalization rates were more than three times higher in LMICs than in high-income countries ^1^. In a previous population-based study in the USA during the 2002–2003 and 2003–2004 influenza seasons, the average annual influenza-associated hospitalization rate among infants younger than 6 months old was 450/100,000 infants compared with 90/100,000 among children 6–23 months old and 30/100,000 in the 24–59-month age group ^7^. In a recent update of the influenza activity in the USA during the 2016–2017 influenza season, 18,184 laboratory-confirmed influenza-associated hospitalizations were reported, and the cumulative incidence among children younger than 5 years old was 44/100,000 children ^41^.

Although influenza-associated hospitalizations are important, outpatient visits are far more frequent in all age groups. In infants, influenza infection tends to be more severe and more often requires hospitalization compared with older patients. Annual influenza-attributable outpatient visit rates in the USA in comparable age groups were approximately 10-, 100-, and 250-fold higher than hospitalization rates for children younger than 6 months, 6–23 months of age, and 24–59 months of age, respectively, in 2002–2004 ^7^.

Global pediatric mortality estimates suggest that the proportion of influenza-associated deaths is highest among those younger than 1 year of age and that 2.8% of all deaths in this age group worldwide are caused by influenza ^42^. Furthermore, it has been estimated that, in 2008, 28,000–111,500 deaths in children younger than 5 years old were due to influenza-associated acute lower respiratory infections, 99% of which occurred in LMICs ^43^. No global mortality estimates, specifically for the subgroup younger than 6 months old, are available, but a study from the USA reported 0.88 deaths associated with laboratory-confirmed influenza per 100,000 infants younger than 6 months old in 2003–2004 ^32^. Moreover, since 2004, when influenza-associated pediatric mortality became a notifiable condition in the USA, the total number of influenza-associated pediatric deaths per season has ranged from 37 to 171, excluding during the 2009 H1N1 pandemic (358 pediatric deaths were reported from April 2009 to October 2010) ^32, 41^.

## Influenza vaccination during pregnancy

Immunological and physiological changes that occur during pregnancy to allow the allograft fetus to survive may affect the immune responses to vaccines and to infection ^44^. Nevertheless, the immunogenicity of IIV, based on the induction of hemagglutinin antibodies, is generally similar in pregnant and non-pregnant women ^45, 46^.

Maternal antibodies against several antigens, from natural infections or vaccination, are selectively transported across the placenta to the fetal circulation ^47^. This vertical antibody transfer is restricted to immunoglobulin G (IgG), and different IgG subclasses are transported with differing efficiency: IgG1 is the most efficiently transported and IgG2 is the least ^48^. IIV during pregnancy boosts the maternal antibody levels and increases the number of antibodies transferred to the fetus ^19, 21, 49^. Because of the theoretical risk to the fetus when the mother receives a live virus vaccine, live attenuated influenza vaccines are not recommended in pregnancy ^23^. The passage of IgGs from the circulation of the mother to the fetus is a selective, cumulative, intracellular process mediated by the neonatal Fc receptor (FcRn) and probably other receptors still to be identified in the syncytiotrophoblast cells in the chorionic villi and subsequent release into the fetal circulation ^50, 51^.

The timing of vaccination during pregnancy influences the quantity of antibodies transferred to the fetus. Transplacental transfer of maternal antibodies begins during the second pregnancy trimester (around 17 weeks of gestation) and peaks towards the end of pregnancy when the expression of FcRn increases ^52^; therefore, antibody concentrations are generally lower in premature than in full-term newborns ^53, 54^. Nevertheless, a study in Switzerland has shown that receipt of IIV at any time during the second and third trimester of pregnancy, but at least 2 weeks before delivery, increased the influenza-specific hemagglutinin antibodies in the newborns ^55^. Similarly, a large IIV clinical trial in pregnant women in South Africa showed that higher transplacental antibody transfer was associated with longer periods of time between vaccination and delivery ^53^. Although the transfer of antibodies is highest later in pregnancy, these results suggest a cumulative transfer and consequently that pregnant women should be vaccinated as soon as IIV is available at any time during pregnancy, not only to protect themselves as early as possible during the influenza season but also to increase the level of antibodies transferred to the fetus.

As shown in the Swiss study and previous studies, vaccination during pregnancy results in a detectable rise in the infant’s antibody levels to the vaccine antigens starting approximately 2 weeks after vaccination ^52, 55^. Owing to the active transport process, fetal hemagglutinin antibody concentrations can equal that of the mother and in some cases exceed maternal levels at term. A wide range of ratios of antibodies in infant at birth to mother at delivery have been reported after influenza vaccination during pregnancy ^19, 49, 56, 57^. Studies that evaluated the immunogenicity of inactivated monovalent pandemic H1N1 influenza vaccines in pregnant women ^56, 57^ normally detected higher newborn–to–maternal ratios of hemagglutinin antibody (1.4 to 2.9) compared with the trials that used trivalent vaccines, where ratios of hemagglutinin antibodies for the different influenza vaccine antigens ranged from 0.7 to 0.8 in the South African trial ^19^ and from 0.8 to 1.1 in a trial from Bangladesh ^49^.

### Prevention of influenza infections

Four randomized controlled trials from Bangladesh ^22^, South Africa ^19^, Mali ^21^, and Nepal ^20^ have demonstrated the efficacy of seasonal IIV vaccination during pregnancy in preventing laboratory-confirmed influenza in infants younger than 6 months old. In the first trial, conducted in Bangladesh from 2004 through 2005, 340 pregnant women received either pneumococcal polysaccharide vaccine or IIV during their third trimester. In that study, vaccine efficacy against laboratory-confirmed influenza in the infants was 63% (95% confidence interval [CI] 5%, 85%); in addition, there was a significant reduction in respiratory illnesses with fever in the infants, and vaccine efficacy was 29% (95% CI 7%, 46%) ^22^. The South African trial included 2,116 human immunodeficiency virus (HIV)-uninfected pregnant women in the second or third trimester who were randomly assigned to IIV or placebo in 2011 or 2012. During the total follow-up period (until the infants reached 24 weeks of age), vaccine efficacy against laboratory-confirmed influenza in the infants was 49% (95% CI 12%, 70%) ^19^. In Mali, 4,193 third-trimester pregnant women were randomly assigned to receive IIV or quadrivalent meningococcal vaccine from 2011 to 2013. During the entire study period, an overall vaccine efficacy of 33% (95% CI 4%, 54%) was demonstrated in infants younger than 6 months old ^21^. The placebo randomized controlled trial in Nepal enrolled 3,693 pregnant women (17–34 weeks’ gestation) from 2011 through 2013, and infant vaccine efficacy was 30% (95% CI 5%, 48%) ^20^. In a recent meta-analysis of the four trials, we calculated that the pooled efficacy of maternal IIV vaccination in preventing laboratory-confirmed influenza in infants younger than 6 months old was 36% (95% CI 22%, 48%) ^58^.

Moreover, three observational studies—two from the USA ^59, 60^ and one from England ^61^—evaluated the effect of IIV administered during pregnancy in preventing medically attended laboratory-confirmed influenza in infants younger than 6 months old. These studies reported a significant protective effect that was generally higher than reported in the clinical trials (71% to 41% reduction in the risk of laboratory-confirmed influenza for infants born to vaccinated women). The pooled effect of all clinical trials and observational studies was 48% (95% CI 33%, 59%) against influenza-confirmed infection among infants whose mothers were vaccinated during pregnancy ^58^.

As mentioned above, outpatient visits are the most frequent medically attended events associated with influenza infection. But to measure the impact of influenza vaccination during pregnancy on hospitalizations that could be precipitated by influenza infection is of crucial importance. Four observational studies reported on the impact of maternal vaccination on laboratory-confirmed influenza hospitalizations in infants ^59, 61– 63^. Although there are a number of challenges regarding observational studies, the pooled vaccine effectiveness of influenza vaccination during pregnancy in preventing infant laboratory-confirmed influenza hospitalizations was 72% (95% CI 39%, 87%) ^58^. The four randomized controlled trials performed to date were not designed to have individual power to detect an effect of maternal vaccination on hospitalizations for laboratory-confirmed influenza. However, in a *post hoc* analysis of the South African trial, maternal vaccination was associated with a 57% (95% CI 7%, 81%) reduction in all-cause acute lower respiratory tract infection (LRTI) among infants younger than 3 months old. Notably, this observation was independent of identifying influenza virus among the hospitalized cases, suggesting that preventing influenza infection may prevent subsequent LRTI in young infants. The negative polymerase chain reaction (PCR) results for influenza infection among these LRTI hospitalized cases may be partly explained by inadequate sample or imperfect test sensitivity. At least in adults, it has been shown that serologic assays demonstrated higher seropositivity for influenza than molecular testing, suggesting higher exposure than when looking at PCR-confirmed illness only ^64^. It is also likely that exposure to the influenza virus initiated a causal chain of events leading to hospitalization for LRTI, even after virus shedding had ceased. Influenza virus infection could have increased the susceptibility to new bacterial nasopharyngeal acquisition, as well as increased the density of existing colonizing bacteria, with disease from these bacteria manifesting only a few weeks after virus clearance ^65– 67^; this is supported by epidemiological studies during the influenza epidemics and animal challenge models that demonstrated that influenza virus infection can enhance the susceptibility to infection with bacteria ^68, 69^.

### Duration of protection

The duration of passive protection in the infant conferred by vaccination during pregnancy depends on the levels of antibodies achieved by vaccination, which are contingent on the efficiency of the transport across the placenta and how quickly the passively acquired antibodies wane. Half-lives of 42–50 days have been described in South Africa ^53^ and Bangladesh ^49^ for the different influenza-specific hemagglutinin antibodies in infants whose mothers received influenza vaccine during pregnancy. However, in HIV-uninfected women vaccinated during pregnancy, half-lives of the antibodies were about 100 days ^53^. The effect of the decay in antibodies in infants was evident from the highest vaccine efficacy observed during the first 8 weeks of life in the South African trial (86% in infants younger than 8 weeks old versus 49% in infants younger than 6 months old) ^70^ and the first 4 months of life in Mali (68% in infants younger than 4 months old versus 33% in infants younger than 6 months old) ^21^.

It has been postulated that the main mechanism of infant protection against laboratory-confirmed influenza infection provided by maternal vaccination is via the IgG antibodies that cross the placenta; however, the overall effect of protection is further confounded by the possible benefits of breastfeeding and reduced risk of exposure to the influenza virus from vaccinated mothers.

### Breastmilk and protection against influenza

Besides the serum antibodies received via the placenta, protection might be provided by antibodies in the breastmilk of vaccinated mothers. This route of protection might be especially important for prematurely born neonates who had limited transplacental transfer of antibodies. However, immune responses in breastmilk after maternal vaccination have been less studied. In the trial in Bangladesh, breastmilk samples were obtained and specific IgA antibodies to H1N1 were significantly higher in IIV recipients than in mothers who received the pneumococcal vaccine for at least 6 months postpartum ^71^. In the same study, exclusive breastfeeding was associated with a decrease in the number of episodes of respiratory illness with fever in infants of mothers who received influenza vaccine during pregnancy.

### Influenza vaccine and HIV-infected pregnant women

Infants born to HIV-infected mothers, even if themselves not HIV infected (HIV exposed, uninfected infants), have an increased risk of hospitalization and death from respiratory virus-associated LRTI, including influenza virus ^72, 73^. In South Africa during 2011, a smaller immunogenicity trial was performed in 194 HIV-infected pregnant women. Women were randomly assigned to receive IIV or placebo, and their infants were followed for laboratory-confirmed influenza until 6 months of age ^19^. Before vaccination and 1 month post-vaccination, HIV-infected compared with HIV-uninfected pregnant women had lower levels of influenza-specific hemagglutinin antibodies and a decreased likelihood of seroconversion ^53^. The transplacental antibody transfer was similar in the HIV-infected and HIV-uninfected cohorts for two of the three vaccine strains, but owing to the lower antibody levels post-vaccination among the HIV-infected women, their newborns had lower antibodies at birth than those born to HIV-uninfected women ^53^. The study in HIV-infected women was not powered to detect vaccine efficacy in the HIV-exposed infants, and a similar influenza attack rate was detected in infants born to mothers who received IIV during pregnancy (5%) and those born to placebo recipients (6.8%; vaccine efficacy: 26.7%, 95% CI −132, 77). However, these rates were higher than those detected in HIV-unexposed infants (1.6% in the IIV group versus 3.6% in the placebo group) ^19^. To date, no other study has explored the benefit of maternal influenza vaccination in HIV-exposed infants in protection against laboratory-confirmed influenza.

## Safety of maternal influenza vaccination

The WHO Global Advisory Committee on Vaccine Safety, in a safety review of the available evidence up until May 2013 of IIV during pregnancy, found no evidence of adverse pregnancy outcomes from vaccination ^25, 74^. The different clinical trials on maternal influenza vaccination also reported rates of adverse events balanced between IIV and control groups. Recent systematic reviews of vaccine safety studies support the conclusions that there is no increased risk in the evaluated adverse outcomes in the mother, the fetus, and the newborn associated with influenza vaccination during pregnancy ^75– 78^. Most of the data, however, were obtained from studies with vaccination in the second and third trimesters. Pharmacovigilance programs need to continue monitoring the safety of maternal vaccination at any stage during pregnancy and, if possible, the impact of vaccination on the long-term development of the children.

## Antivirals

In December 2012, the US Food and Drug Administration expanded the use of the antiviral oseltamivir phosphate to include infants 2 weeks of age and older for the treatment of uncomplicated influenza ^79^. This is the only antiviral approved for the treatment of influenza in infants but is not indicated for prevention in those younger than 1 year of age, although it can be used prophylactically in children older than 1 year of age at high risk, including those in close contact with individuals with known or suspected influenza infection ^80^.

## Future considerations

Since influenza vaccination during pregnancy was demonstrated to confer some protection to both mothers (not discussed in this review ^19, 21^) and young infants against influenza infection, this strategy should be used as the first line of protection. Nonetheless, there are still some considerations and open questions for future research in this field. These include the need for more immunogenic influenza vaccines for pregnant women to increase the concentration of the antibodies transferred transplacentally, which would last for a longer period in the infants. Infant passive protection beyond the first 2–4 months of life is desirable, as only after 6 months of age are infants eligible to receive influenza vaccine. More immunogenic vaccines could probably be achieved with higher antigen concentrations or the use of adjuvants. During the 2009 H1N1 pandemic, newer formulations of inactivated antigens administered with adjuvants were found to enhance the immune responses compared with non-adjuvanted vaccines ^81– 85^. Based on the immunogenicity data from the two South African trials, despite vaccine efficacy observed in HIV-infected mothers (which was similar to HIV-uninfected women, not discussed in this review), alternate strategies to protect the HIV-exposed infants need to be considered ^19^. Whilst vaccine-induced cell-mediated immunity might play a role in adults, protection in the infants will be mediated only by the presence of antibodies; hence, again, higher antibody concentrations need to be elicited in the women to prevent disease in their HIV-exposed infants.

The development of universal vaccines containing common antigens that could provide protection against different virus strains, reducing the need for annual vaccine update, and hopefully provide protection across multiple pregnancies is also being explored.

The role of breastmilk antibodies in protection against influenza illness in infants also needs further elucidation.

Furthermore, evidence regarding an increased susceptibility or severe disease outcome in young infants born to influenza virus-infected mothers is still to be shown. Animal models such as a murine pregnancy model which mimics key clinical findings of the 2009 H1N1 influenza pandemic ^86^ could help demonstrate any causal relationship between maternal infection during pregnancy and offspring’s disease outcome.

The paucity of high-quality data on the effect of vaccination during pregnancy against hospitalizations associated with laboratory-confirmed influenza in infants is still a limitation for truly estimating the effect size of this intervention on more severe outcomes. Large prospective multi-season studies with long-term follow-up powered to address this endpoint should be performed. Further research is also needed to better define the burden of influenza in young children, especially those presenting with non-respiratory symptoms or those who might have cleared the virus at the time of laboratory testing, using vaccine probe as a strategy to delineate this, as done in the South African trial ^70^.

## Abbreviations

CI, confidence interval; FcRn, neonatal Fc receptor; HIV, human immunodeficiency virus; IgG, immunoglobulin G; IIV, inactivated influenza vaccine; LMIC, low- to middle-income country; LRTI, lower respiratory tract infection; PCR, polymerase chain reaction; WHO, World Health Organization.
